# Use of topical colistin in bacterial keratitis caused by extensively drug-resistant Pseudomonas Aeruginosa: a case report

**DOI:** 10.5935/0004-2749.20230039

**Published:** 2023

**Authors:** Jesus Maticorena-Quevedo, Lía Patiño-Valderrama, Karin Arellano-Caro

**Affiliations:** 1 Department of Ophthalmology, Hospital Nacional Edgardo Rebagliati Martins, Lima, Peru.; 2 Cornea and External Diseases Unit, Department of Ophthalmology, Hospital Nacional Edgardo Rebagliati Martins, Lima, Peru.

**Keywords:** Keratitis, Eye infection, bacterial, Drug resistance, Pseudomonas infection, Colistin, Genes MDR, COVID-19, Ceratite, Infecção ocular bacteriana, Resistência a medicamento, Infecção por pseudomonas, Colistina, Genes MDR, COVID-19

## Abstract

Bacterial keratitis caused by multidrug-resistant strains of *Pseudomonas aeruginosa* is a therapeutic challenge due to a limited number of active antimicrobials and rapid progression to corneal necrosis and perforation. To report the use of topical colistin and surgical tarsorrhaphy in a case of keratitis caused by extensively drug-resistant *Pseudomonas aeruginosa* in a patient with severe coronavirus disease-2019 (COVID-19) pneumonia. A 56-year-old male was admitted to the intensive care unit with clinical symptoms of severe COVID-19 pneumonia. During his stay in the unit, he developed rapidly progressive keratitis with *Pseudomonas aeruginosa* resistant to all drugs except for colistin on culture. Due to incomplete lid closure, a temporary tarsorrhaphy was performed, and a regimen of descending-dose topical colistin was initiated. After five weeks, keratitis resolved completely. Extensively drug-resistant *Pseudomonas aeruginosa* is an unusual cause of bacterial keratitis. We describe the safe and effective use of topical colistin in a case with severe corneal involvement.

## Abstract (pt)

A ceratite bacteriana causada por cepas multirresistentes de *Pseudomonas aeruginosa* é um desafio terapêutico, devido à disponibilidade limitada de antimicrobianos e à rápida progressão para necrose e perfuração da córnea. O objetivo deste artigo é relatar o uso de colistina tópica e tarsorrafia cirúrgica em um caso de ceratite por *Pseudomonas aeruginosa* amplamente resistente a medicamentos em um paciente com pneumonia grave por COVID19. Um homem de 56 anos foi internado em uma unidade de terapia intensiva com sintomas clínicos de pneumonia grave por COVID19. Durante sua permanência na unidade de terapia intensiva, o paciente desenvolveu uma ceratite rapidamente progressiva, cuja cultura foi positiva para *Pseudomonas aeruginosa* resistente a todos os antimicrobianos, exceto colistina. Devido ao fechamento incompleto da pálpebra, foi realizada uma tarsorrafia temporária e foi instituído um esquema de colistina tópica em doses decrescentes. Após cinco semanas, a resolução completa da ceratite foi alcançada. *Pseudomonas aeruginosa* amplamente resistente a medicamentos é uma causa incomum de ceratite bacteriana. Este relato descreve o uso seguro e eficaz da colistina tópica em um caso com comprometimento corneano grave.

## INTRODUCTION

Bacterial keratitis represents the fourth most common cause of blindness worldwide, having the highest incidence in low-income countries^(1)^. Among the main bacterial isolates, *Pseudomonas aeruginosa* (PA) is res­ponsible for the majority of severe clinical presentations, characterized by rapid progression. Likewise, according to the World Health Organization (WHO), this pathogen is currently considered priority one in the list of bacteria for which new antibiotics are urgently needed^(2)^. Because of this, the management of bacterial keratitis caused by PA is extremely complex, especially if multidrug resistance is present.

We present the case of a 56-year-old man with severe pneumonia due to coronavirus disease-2019 (COVID-19) hospitalized in an intensive care unit (ICU), who developed a severe corneal ulcer due to extensively drug-resistant PA (XDR-PA). We report the use of topical colistin with satisfactory outcomes.

## CASE REPORT

**Figure 1:**
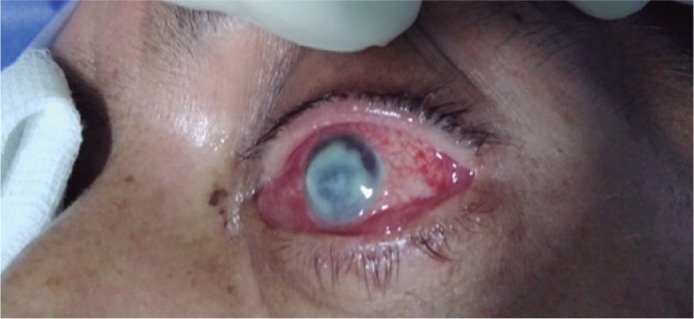
Patient during the first ophthalmological evaluation. Eyelid thickening, diffuse bulbar conjunctiva hyperemia, and chemosis are observed. The corneal ulcer presents as a whitish lesion with a ring-like stromal infiltrate that covers 90% of the corneal tissue and involves the lower limbus.

A 56-year-old man was admitted to the ICU due to severe COVID-19 pneumonia. Two months after the admission, the ophthalmology department was consulted for a recent “whitish” lesion in the left eye. Both eyes showed incomplete lid closure, predominantly in the left eye, accompanied by slight proptosis, edema, and erythema in both eyelids. The corneal ulcer presented as a whitish lesion of 9 x 9 mm with irregular borders and a ring-like stromal infiltrate, including the lower limbus. Seropurulent discharge in the conjunctival fornix and 2 mm hypopyon was seen (Figure 1). After the initial evaluation, fortified antibiotics of ceftazidime 50 mg/ml and vancomycin 50 mg/ml were administered every hour. Complementary images were requested, a brain and orbital computed tomography scan showed a slight increase in the volume of the preseptal tissues without hyperdense areas in the vitreous cavity of the left eye. Because of the patient’s hemodynamic instability, it was not possible to take a sample for a culture of the lesion at that moment.

Four days after the initial evaluation, the corneal infiltrate did not show significant changes. Because of the improvement of the clinical status, corneal scarping cultures of the entire edge and base of the ulcer were taken. After 72 hours, the culture and antibiogram were positive for PA resistant to all antibiotics except colistin, with a minimum inhibitory concentration (MIC) of <2 (Table 1).

**Table 1:** Antibiogram of the corneal scraping culture Results of the corneal scraping culture: *Pseudomonas aeruginosa*

Antimicrobial	MIC	Interpretation
Amikacin	>32	R
Aztreonam	>8	R
Cefepime	>8	R
Ceftazidime	>16	R
Ciprofloxacin	>2	R
Colistin	<2	S
Gentamicin	>8	R
Imipenem	>8	R
Levofloxacin	>4	R
Meropenem	>8	R
Piperacillin/Tazobactam	64	I
Tobramycin	>8	R

MIC= Minimum inhibitory concentration; R= Resistant; S= Sensible; I= Intermediate.

**Figure 2:**
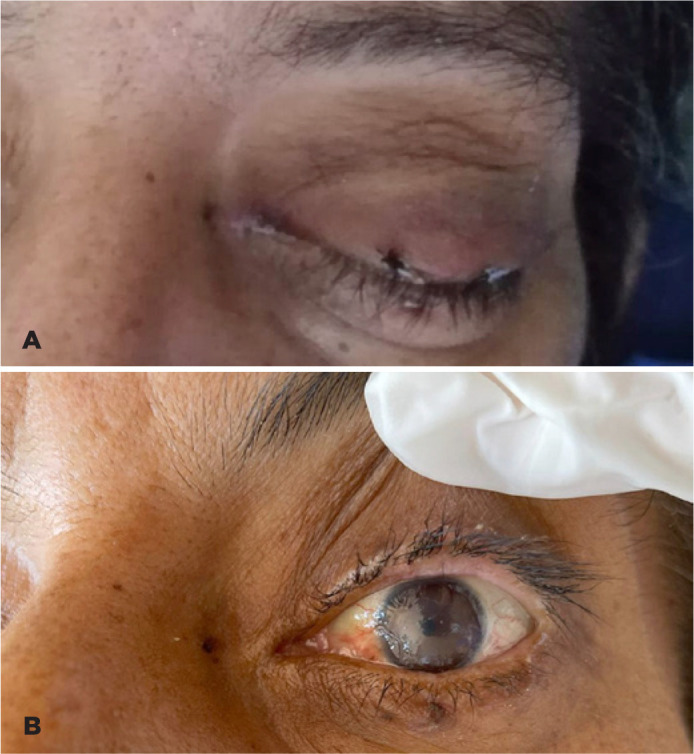
A. Temporal tarsorrhaphy was performed on the seventh day after the onset of symptoms. From this day, topical colistin was instilled through the medial canthus. B. Clinical photograph of the patient 6 weeks after the onset of symptoms and 5 weeks after the use of topical colistin and temporary tarsorrhaphy. A slightly opaque corneal tissue is observed, but with a total resolution of the corneal infiltrate and without areas of thinning.

Due to the culture result, the rapid clinical evolution and the persistence of incomplete lid closure despite the marked decrease in soft tissue edema in the left eye, it was decided to perform a temporary tarsorrhaphy (Figure 2A), and prescribe topical colistin. Seven days after the first evaluation, topical colistin every 2 hours was initiated in the left eye.

Five weeks from the start of topical colistin, physical examination revealed opaque corneal tissue without ulcerative/infiltrative lesions or thinned areas (Figure 2B). Because of the patient’s clinical condition, it was not possible to perform the visual acuity assessment or the slit-lamp ophthalmological examination at any time. However, with topical colistin and temporary tarsorrhaphy, the patient showed excellent improvement of XDR-PA corneal ulcer.

## DISCUSSION

According to the international consensus of acquired microbial resistance, multidrug-resistant PA infection (MDR) is defined as an infection caused by PA that has resistance to three different families of antimicrobials. XDR is an infection only sensitive to one or two families of antibiotics, and pan-drug-resistant (PDR) infection is resistant to all antimicrobial families^(3)^. The prevalence of antibiotic resistance varies according to the region where the bacteria have been isolated. Up to 16.67% of PA positive cultures are XDR or PDR^(4)^. Definitely, the emergence of resistant strains is extremely concerning due to the limited availability of antibiotics with good corneal penetration^(5)^.

The resistance mechanisms are very complex and are due to both chromosome-encoded resistance genes and the ability to acquire mobile genetic elements (MGEs) from intra- and interspecies transfer of antimicrobial resistance.^(6)^ Therefore, plasmids, transposons, and integrons are some examples of MGEs that allow the bacteria to survive adverse conditions. MGEs act as vectors for the dissemination and capture of resistance genes in a mechanism called horizontal gene transfer^(6,7)^. These mechanisms, alongside others, such as the formation of biofilms and mutations in the quinolone resistance-determining regions, are some examples of how antimicrobial resistance develops in ocular infections caused by PA^(6)^.

It has been shown that most cases have corneal infiltrates greater than 6 mm that double their size in 24 hours and more than 80% of patients remain with visual acuity worse than 20/200^(8)^. In our patient, despite the severe compromise and depth of the infiltrate, perforation of the corneal tissue was avoided with the prolonged use of topical colistin and surgical occlusion of the eyelid.

Colistin is a polymyxin antibiotic, which use has reemerged due to the increase in resistant strains and the lack of availability of new antimicrobials^(4)^. The mecha­nism of action has not been fully elucidated, but it is known that it acts on lipid A of the lipopolysaccharides of the cell wall and increases cellular permeability through its detergent action for the entry of the same molecule in what is known as a ‘’self-promoted uptake’’ pathway^(4)^. Furthermore, it promotes the production of reactive oxygen species and the release of cytokines, such as tumor necrosis factor-alpha (TNF-α) and interleukin 8 (IL-8)^(4)^.

The use of topical colistin for infectious keratitis was first described in 1969 and has had mostly satisfactory results in the case of resistant PA strains^(5,8-10)^. In this way, Jain et al. demonstrated that the use of 0.19% topical colistin allowed total or partial (requiring cyanoacrylate) recovery in half and one-third of patients with MDR-PA keratitis, respectively^(9)^. However, 1.6% colistin use has also been reported with success, considering that only a dilution period is required in the preparation and that the higher concentration would presumably have greater efficacy^(8)^. In our case, due to the greater number of satisfactory reports with a lower concentration and absence of local adverse effects mentioned, it was decided to use the 0.19% topical colistin regimen. The final outcome was very successful.

Finally, we report the case of a male patient with multiple complications derived from severe pneumonia due to COVID-19, who developed a severe corneal ulcer in the left eye secondary to XDR-PA, which was managed with topical colistin and temporal tarsorrhaphy. We describe the safe and effective use of this antimicrobial with complete resolution after five weeks of treatment.
